# Open Joint

**Published:** 1898-04

**Authors:** J. C. Michener

**Affiliations:** Colmar, Pa.


### OPEN JOINT.

By J. C. Michener, V.S.,
COLMAR, PA.

This case is reported to show what may be done with an antiseptic blister.

On February 5th a large, spirited, young horse broke loose and ran upon a ribbon, or flat-wire, fence. Striking it obliquely, his right front ankle slid upon the top of a wire, cutting the inside of the joint open its entire width from below upward. A neighbor, who makes and sells a horse liniment, was called and succeeded in arresting the hemorrhage by saturating cotton with his liniment and retaining by a firm bandage. Not much lameness at the time, and a quick recovery promised. By February 9th, when I was called, the horse refused to place any weight upon the foot and kept it in almost constant motion, quivering and sweating and refusing to eat.

The synovia had trickled down over the hoof, congealing, like an ice-fall, reaching from the wound to the ground. The parts were thoroughly cleansed by a 10 per cent. chloride of zinc lotion, and the hair clipped from the entire joint. The blister ointment was well rubbed in and the wound plastered shut. Then a piece of muslin to envelope the joint was spread with a liberal coat of the ointment and retained by bandage.

In twenty-four hours found the horse resting his weight upon the injured limb, feeding, and looking quite cheerful. Allowed the blister and bandage to remain another twenty-four hours, when they were removed and the parts cleansed. The wound swollen shut. *No synovial discharge.*

Turned him in loose box-stall. Did well for four days, when he again manifested pain, and the next day the wound opened in the centre, the size of a straw, from which the synovia spurted when he moved. Applied the blister as upon the first day and allowed it to remain forty-eight hours. Had no more synovial discharge or lameness. The horse was put to work upon February 18th, and remains none the worse of the accident.

